# Supplementary figures and images for: Cross-reactivity of rPvs48/45, a recombinant Plasmodium vivax protein, with plasma from Plasmodium falciparum endemic areas of Africa
Source: PLoS One. 2025 Mar 18;20(3):e0302605. doi: 10.1371/journal.pone.0302605 (PMC11918314; doi:10.1371/journal.pone.0302605)

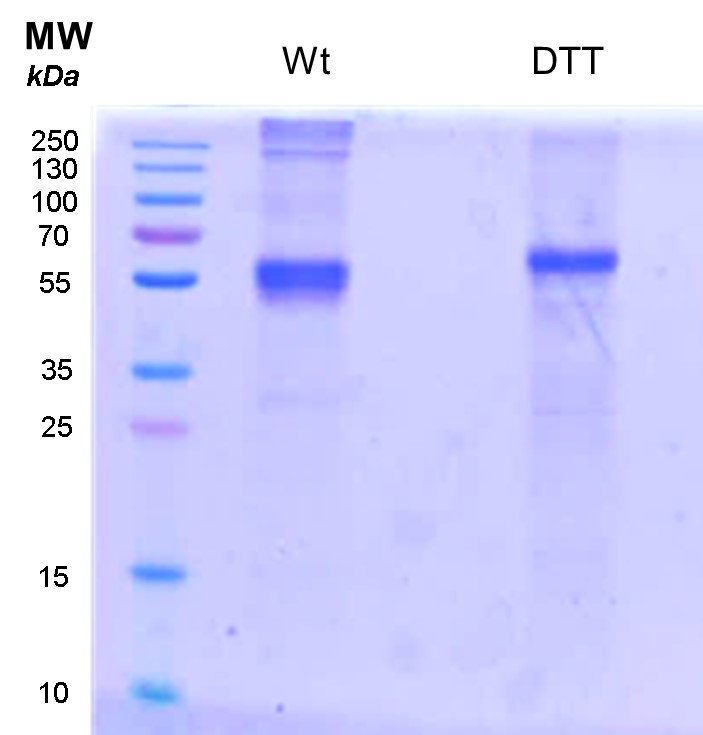

Supplement: S1 Fig — CHO-rPvs48/45 protein identity was confirmed using 12% SDS-PAGE gel in western blot. Analysis was carried out under reducing (0.05 mol/L dithiothreitol, DTT) and non-reducing conditions (wt) [22]. (TIFF) [file pone.0302605.s001.tiff]

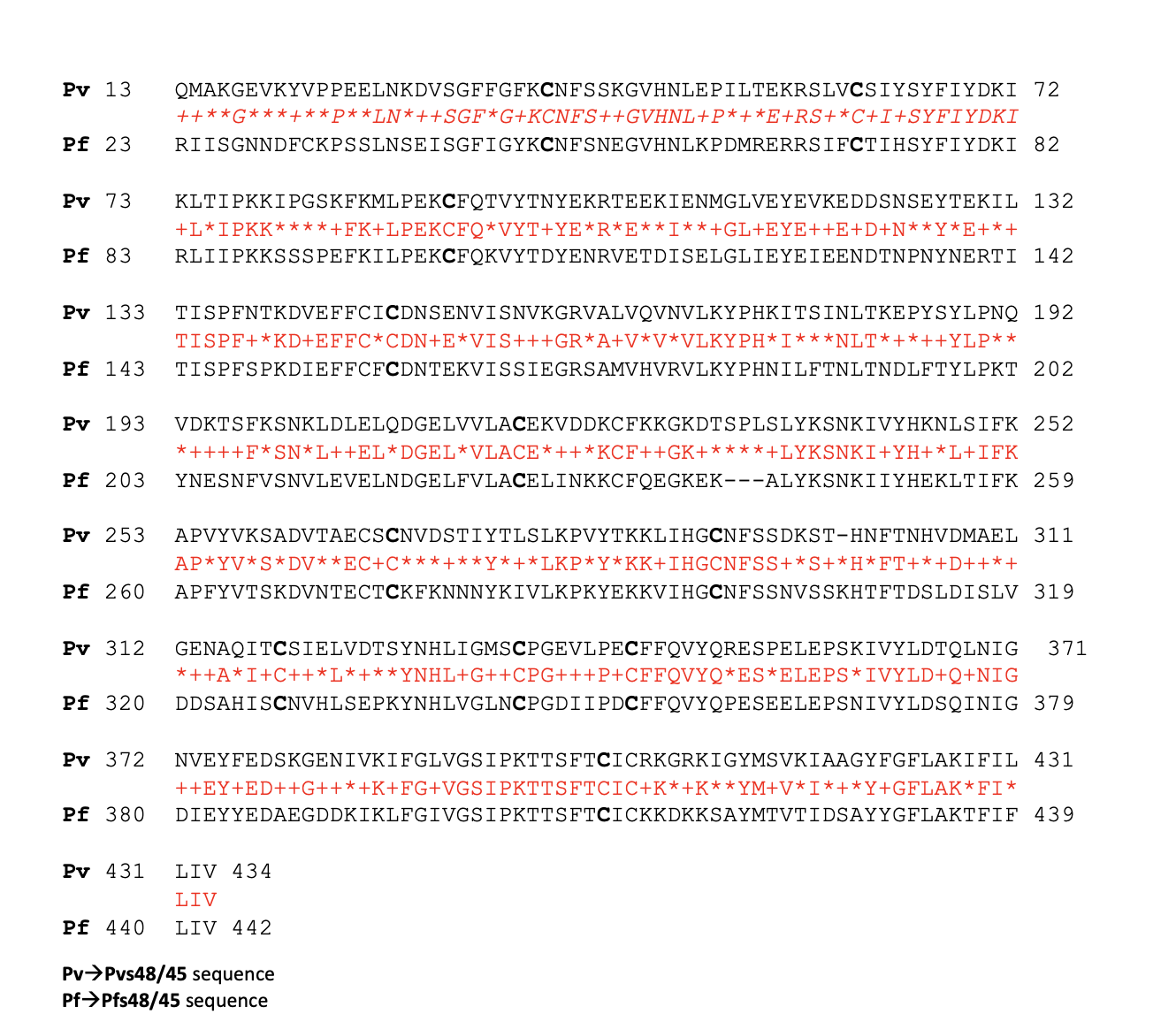

Supplement: S2 Fig — The amino acid (aa) sequences alignment of full-length Pvs48/45 and Pfs48/45 proteins were obtained using the PlasmoDB database, and sequences matched with Blastp (protein-protein BLAST; https://bit.ly/3C0hPpK). Pfs48/45 and Pvs48/45 share ~56% identity (238 out of 423) and ~ 78% similarity in their protein sequences. Conserved cysteine residues are identified by the C letter in black and bold. In the red sequence, identical amino acid residues are identified by (letter|), the similar residues by (+) and the different residues by (*). (TIFF) [file pone.0302605.s002.tiff]
