# Supplementary material for: Cross-reactivity of rPvs48/45, a recombinant Plasmodium vivax protein, with plasma from Plasmodium falciparum endemic areas of Africa
Source: PLoS One. 2025 Mar 18;20(3):e0302605. doi: 10.1371/journal.pone.0302605 (PMC11918314; doi:10.1371/journal.pone.0302605)
Supplement: S1 Checklist — (DOCX) [file pone.0302605.s003.docx]

*PLOS ONE* Humane Endpoints Checklist

*PLOS ONE* manuscript number: ________ **PONE-D-24-12287**_______________

**Complete the following if your study design includes death of a regulated animal as a likely outcome or planned experimental endpoint. Please also include all information in the Methods section of your manuscript.**

**ITEM 1.** **Describe whether humane endpoints* were used for all animals involved in the study.**

|  | **Recommendation** | **Section/Paragraph** |
| --- | --- | --- |
| **If humane endpoints* were used, report the following:** | | |
| **1** | **The specific criteria used to determine when animals should be euthanized** | **The overall decision to euthanize the animals as an endpoint in the study, was based on the scientific justification, humane considerations, pre-established criteria, ethical guidelines, veterinary oversight and regulatory compliance.** |
| **2** | **Once animals reached endpoint criteria, the amount of time elapsed before euthanasia** | Once endpoint criteria were reached, animals remained in captivity for less than a (1) week.  Mice were under partial anesthesia using isoflurane 1,5 liters/minute. After the final readout, mice were euthanized by 5 minutes of CO_2_ direct exposure followed by cervical dislocation, according to the American Veterinary Medical Association (AVMA) guidelines. |
| **3** | **Whether any animals died before meeting criteria for euthanasia** | **No** |
| **If humane endpoints* were not used, report the following:** | | |
| **1** | **A scientific and ethical justification for the study design, including the reasons why humane endpoints could not be used, and discussion of alternatives that were considered but could not be used** | **NA** |
| **2** | **Whether the institutional animal ethics committee specifically reviewed and approved the anticipated mortality in the study design** | **In accordance with ethical guidelines and regulatory requirements, the institutional animal ethics committee thoroughly reviewed and approved all aspects of the study design, including anticipating mortality. The Committee carefully assessed the scientific justification for any anticipated mortality, ensuring that it was necessary for achieving the research objectives and advancing scientific knowledge while minimizing harm to the animals involved. Additionally, the committee evaluated measures in place to mitigate risks and ensure animal welfare throughout the study, including appropriate euthanasia criteria procedures. The approval from the institutional animal ethics committee underscores our commitment to upholding the highest standards of animal welfare and ethical conduct in research.** |

**ITEM 2.** **Include the following details of the study design and outcomes.**

|  | **Recommendation** | **Section/Paragraph** |
| --- | --- | --- |
| **1** | **The duration of the experiment** | **45 days** |
| **2** | **The numbers of animals used, euthanized, and found dead (if any); the cause of death for all animals** | In the study, a total of twelve male and female BALB/c mice, aged 6-8 weeks old, were utilized, with six assigned to the experimental group and six control group.  Throughout the study, detailed records were maintained regarding the number of animals used, euthanized, and any found deceased. |
| **3** | **How frequently animal health and behavior were monitored** | **Monitoring of animal health and behavior was assessed through daily observations to assess general health, activity levels, food and water consumption, and any sign of distress or illness. Additional checks were performed before and after interventions, to ensure the well-being of the animal.** |
| **4** | **All animal welfare considerations taken, including efforts to minimize suffering and distress, use of analgesics or anaesthetics, or special housing conditions** | **Comprehensive measures were implemented to uphold animal welfare throughout the study. Efforts to minimize suffering and distress were prioritized at every stage of the research process. This included the careful design of experimental procedures to minimize pain and discomfort, as well as the provision of appropriated analgesic or anesthetics when necessary to alleviate any potential distress. Additionally, special housing conditions were provided as needed to ensure the comfort and well-being of the animals, taking into account factors such as social interaction, environmental enrichment, and species-specific requirements. Regular monitoring was conducted for early detection of any signs of distress or adverse effects. No veterinarian interventions were required, as there were no welfare concerns during the study.** |
| **5** | **Any special training in animal care or handling provided for research staff** | **Research team members and veterinarians underwent specialized training in animal care and handling either through internal hands-on training or through external.** |

***Definition of a humane endpoint**

A humane endpoint is an experimental endpoint at which animals are euthanized when they display early markers associated with death or poor prognosis of quality of life, or specific signs of severe suffering or distress. Humane endpoints are used as an alternative to allowing such conditions to continue or progress to death following the experimental intervention (“death as an endpoint”), or only euthanizing animals at the end of an experiment. Before a study begins, researchers define the practical observations or measurements that will be used during the study to recognize a humane endpoint, based on anticipated clinical, physiological, and behavioral signs. These may include, for instance, body temperature or weight changes, tumor size or appearance, abnormal behaviors, pathological changes, ruffled fur, reduced mobility, body posture, or expression of specific body fluid markers. Please see the NC3Rs guidelines for more information.

**ARRIVE Guidelines**

*PLOS ONE* encourages authors to follow the [Animal Research: Reporting of In Vivo Experiments (ARRIVE) guidelines](http://www.nc3rs.org.uk/arrive-guidelines) for all submissions describing laboratory-based animal research and to upload a completed [ARRIVE Guidelines Checklist](http://www.nc3rs.org.uk/sites/default/files/documents/Guidelines/NC3Rs%20ARRIVE%20Guidelines%20Checklist%20%28fillable%29.pdf) to be published as supporting information.
