# Supplementary material for: Cross-reactivity of rPvs48/45, a recombinant Plasmodium vivax protein, with plasma from Plasmodium falciparum endemic areas of Africa
Source: PLoS One. 2025 Mar 18;20(3):e0302605. doi: 10.1371/journal.pone.0302605 (PMC11918314; doi:10.1371/journal.pone.0302605)
Supplement: Attachment — Submitted filename: Second responses to reviewers.docx [file pone.0302605.s006.docx]

**Letter**

We would like to express our thanks once again to the editor and reviewers for the valuable comments and for giving us a second opportunity to improve the document. The attention paid to the issues they raised has enabled us to improve the manuscript considerably.

Minor change in Figure 1A occurred, with NHS (naive human serum) replaced by NHP (naive human plasma) and the p-value comparing the OD value of NIG versus NHP (negative control) has been inserted.

For the rest, we provide below a detailed point-by-point response raised by reviewer 1.

We hope that the manuscript is now suitable for publication and remain at your disposal for any further questions.

PS: Please note that the indications of lines given in our responses are in accordance with the revised clean version of the manuscript.

Sincerely yours

Saidou Balam

December 20, 2024

**Responses to Reviewer Comments**

**1.** The Literature should remain focused on the target antigen and the intent should not be to review the literature on P. vivax vaccines. This will focus on the manuscript and also help remove unnecessary too many self-citations.

**Response (R)-**	*We agree with your suggestion and most of the references not directly related to TB were deleted except those on vaccine necessary for a paragraph description.*

**2.** Why are there are no co-authors who collected and provided sera from TZ and NIG?

***R-***	*Dr. Sope Olugbile, co-author of this study, contributed with samples from the NIG. From TZ, nobody contributed to this publication in a manner acceptable for the international standards of authorship.*

**3.** line 53: Pf IFA data is questionable because the sera likely contained anti-Pf IgM induced by the Pf booster dose and it may not be due to boosted anti-Pvs48/45 abs.

**R-**	*The* *data presented reflects the observed results. Given the high homology of P48/45 in both parasite species, is plausible that the immune system generated a secondary IgG response.*

**4.** Provide a more recent ref for #3 for WHO

**R-**	*This reference was updated to the 2024 World Malaria Report.*

**5.** Many references are irrelevant (in view of my point 1). These include 17-19, 23, 25-27.

**R-**	*Irrelevant references are now removed.*

**6.** Line 123: it should be plasma because the blood was collected in the presence of EDTA.

**R-**	*For human samples collected with EDTA the word “sera” was replaced to “plasma” throughout the manuscript text and Figure (s).*

**7.** lines 137-146: I understand these mice data were published previously but the same mice after rest period were boosted with Pf. In that case lines 137-146 can be simply replaced with a sentence with appropriate reference.

**R-**	*The description has been now summarized (Cf method-mice immunization section: lines 140-150)*

**8.** Line 165xxx: Change to "....The reaction was stopped using ...ul 1 M sulfuric acid and the optical density measured.... was the OD measured at both the wavelengths ?

**R-**	*The description was revised and completed as follow (Lines164-167):*

“*The reaction was stopped using 50mL of 1M sulphuric acid (Merck, 1.00731.1000) and the optical density (OD) was measured at both 450 nm and 630 nm wavelength, with the later used for background correction, using a TECAN Nano Quant Infinit M200 PRO spectrophotometer*”

**9.** Lines 178-183: If these beads were prepared as published then just say so and cite the ref. and delete these lines.

**R-**	*We think it's necessary to maintain a few descriptions of the method. However, the text has been simplified while citing the reference.Lines: 177-190*

**10**. Line 253: You don't know whether the conformation was the same or not. You are only looking at the ag-ab reactivity.

**R-**	*You are correct.* *The sentence was removed (cf. esult section, subtitle a.).*

**11.** In Figs. 1 and 2 when presenting human sera OD values - are these observed or after subtraction of (negative control = 3SD)? If these are after subtraction, then I don't have any further comment. If not, then the right way will be to present net OD after subtraction. In the latter case all further % values and calculations throughout the manuscript are likely to be affected.

**R-**	*We would like to thank the reviewer for this point of view. However, it needs to be noted that the ODs presented here are those without subtraction of the ODs of negative control. We believe that since the 3SD of the control are not representing an internal control for each interest sample, it is desirable to present both the ODs of the samples and the negative control as we have shown in figure 1A. Indeed, the OD of the controls is significantly lower than even the OD of the NIG samples, which is the lowest of the four countries (Cf. graphic, Figure 1A). As regards the calculation of prevalence (positive samples), these 3SD of the control are applied to each sample ( cf. ELISA positivity definition, Lines: 167-169).*

**12**. Sub-heading on lines 274-274: Somehow the use of Concerning is not sitting well in my reading. The authors might want to rephrase.

**R-**	*We have modified the subtitle as follow in Line 271:*

*“Age- and gender-dependent cross-reactivity of the rPvs48/45 protein with P. falciparum African immune plasma”*

**13**. Lines 317-322: Suggest rewrite "...., was then used in SMFA for functional cross-reactivity against Pf gametocytes (Table 1). At the end of this paragraph, the authors should state why they could not those abs against Pv in DMFA.

**R-**	*The section was rewritten as suggested to add this* *sentence:” DMFA with P. vivax could not be performed due to the unavailability of affinity purified antibodies from African plasma, which were denatured because of prolonged storage”. Lines 316-318*

**14**. line 338: replace vigorous with strong.}

**R-**	*The word was modified. Line 333.*

**15.** ...260. Further immunization with a single dose of Pf gametocytes resulted in significant boosting of anti-Pvs48/45 ELISA....

**R-**	*The sentence was modified. Line 337-338.*

**16.** Line 355-357: IFATR concern in view of my comment number 3.

**R-**	*We apologize for the error. The same figure was mistakenly included. The IFAT figure has been replaced (Figure 3B).*

**17**. Line 369: ....collection, confirms previously reported cross reactivity (ref. 50).

**R-**	*The reference was included. Now is Ref 44 (Bansal GP et al). Line 367.*

**18**. Line 375: Move ref. 50 to modified line 369 and ref. 51 is not from mice but humans.

**R-**	*References were modified as suggested Ref 44. Line 367-368. Ref 51. is now not cited as for mouse.*

**19.** Line 397: ...exposed repeatedly. and delete periods as a function of their age from line 398.

**R-**	*The sentence was modified as suggested. Line 394.*

**20**. Line 432: I am not convinced about IFAT data against Pf as stated before.

**R-**	*IFAT figure was replaced. cf figures 3A.*

**21**. Line 434: ...in mice provide further support for Pvs48/45 as a target for TB vaccine.

**R-**	*The sentence was modified*. *Line 428.*
