# Supplementary material for: Cross-reactivity of rPvs48/45, a recombinant Plasmodium vivax protein, with plasma from Plasmodium falciparum endemic areas of Africa
Source: PLoS One. 2025 Mar 18;20(3):e0302605. doi: 10.1371/journal.pone.0302605 (PMC11918314; doi:10.1371/journal.pone.0302605)
Supplement: Attachment — Submitted filename: Cover letter and Responses to reviwers.docx [file pone.0302605.s005.docx]

**Cover letter and Responses to Review Comments to the Author**

We thank the two anonymous reviewers for their valuable comments. Attention to the issues they raised has significantly improved the manuscript. We provide a detailed point-by-point response below each comment.

Sincerely yours

29.11.2024

Saidou Balam

**Responses to Review Comments**

**Reviewer #1:**

This manuscript reports essentially confirmatory data on the recognition of Pvs48/45 by antibodies in people previously exposed to P. falciparum thus confirming cross-reactivity on a larger sample size from 4 different African countries. The authors have cited previously published work on the same phenomenon of cross-reactivity. The work in the cited reference showed >70% cross recognition by sera from a study conducted in Southern African region (Zimbabwe). While the studies and data support the conclusions drawn, I would like to see authors respond to my major and minor concerns listed below:

Comment # 1. Line 61:replace ...,"(exposed only to P. falciparum)".... with ....."exposed predominantly to P. falciparum"...., unless authors have tested blood for infecting parasites. Co-infections with other Plasmodium species are quite common.

***Response #1:*** *The likelihood of having P. vivax circulation in the study areas was mentioned in the summary, introduction and discussion sections.*

Comment # 2. Lines 83-97: should be deleted because they are irrelevant to the studies.

***Response # 2****: We have done extensive editing between lines 77-108*

Comment # 3. The Introduction is very unfocussed and very similar superfluous and needs to be carefully made succinct with the overall goal of studies properly stated with due credit to previously published work.

***Response #3****. The introduction section has been revised to enhance focus and conciseness and adequately establish the study’s objectives in light of previous research.*

Comment # 4. 2.1 and 2.2 have very similar details and efforts need to be made to minimize duplication.

***Response #4.*** *We thank the reviewer for this remark. We have reviewed the sub-chapters and eliminated redundancies.*

Comment # 5. 2.3: Do not include results in the methods.

***Response # 5****: The section now exclusively describes the immunization of mice and sample collection methods, with results moved to the corresponding section.*

Comment # 6. 2.4 subheading can be deleted, and details can simply be stated because they have been published previously and well known at this point.

***Response # 6:*** *This section has been removed, and references to previous studies have been cited in the relevant section of the Results or Discussion.*

Comment # 7. Similarly, 2.5 details are likely published in 62 and be referenced without the need for a sub-heading.

***Response # 7.*** *We have removed the subheading and cited the referenced work.*

Comment # 8. Line 77: what is the superscript 2 on the word derivatives for?

***Response #8.*** *This typo error was corrected.*

Comment # 9. 2.6: Why have authors included Ref number for each chemical from a particular vendor?

***Response # 9****: These references were added to ensure precision, but we acknowledge the potential for redundancy, and they are removed.*

Comment # 10. This reviewer does not like the term competition term for the ELISA. All they are doing is to self deplete cross-reacting antibodies. In the same they include ref. 72. Are any details different from those in the indirect ELISA?

***Response # 10****. We appreciate the feedback. This assay is indeed more accurately described as an auto-inhibition ELISA to deplete cross-reacting antibodies against rPvs48/45. This clarification has been made in the Methods section under the ELISA description.*

Comment # 11. 2.9: Why did they include complement in the SMFA? Does it depend upon complement?

***Response #11.*** *Complement was included based on its role in immune-mediated parasite killing, particularly within transmission-blocking immunity. Complement can enhance antibody efficacy against Plasmodium parasites by facilitating opsonization, membrane attack complex formation, and phagocytosis. Although the SMFA can operate without complement, we included it to explore if it might enhance antibodies' effectiveness against rPvs48/45. While complement is not essential for the assay, this approach allowed us to assess potential synergistic effects on antibody neutralization.*

Comment # 12. Line 361: the reference 81 pointed out above was from a study on sera from Africa and not Latin America and that study too showed >70% sera positive for cross-reactivity.

*Response #12. The original MS had only 70 references. Anyway, the reference to studies in Latin America, specifically in Colombia, was revised and explained further.*

Comment # 13. The studies on *P. falciparum* SMFA with affinity purified are nice and not surprising. What the authors must do is to evaluate the same antibody in DMFA with P*. vivax*. In the absence of this data the present studies are only confirmatory and add incremental knowledge.

***Response #13.*** *We appreciate the reviewer's suggestion and agree that conducting DMFA with P. vivax would provide valuable additional data. Unfortunately, the antibody that had been affinity-purified from African sera in Switzerland became denatured due to long storage in customs and could not be used. We are exploring possibilities for conducting DMFA with P. vivax in future studies.*

Comment # 14: Comments on Figures:

Figures 1,2 and 3: Delete Panel A and simply add % responded column in the table. In fact, I would suggest that modified Figures 1-3 can simply be three panels of a single Figure.

Figure 4: It can be deleted because it does not add any knowledge. Panel A simply shows that BF40 has less antibody than BF 70. Panel B ELISA titration curves for a few select sera. Panel C just shows which eluted fraction has the maximum eluted antibody amount. The entire figure does not contribute anything new.

Figure 5: The authors need to demonstrate IgM (day 270-280) and IgG (days >300) responses against *Pfs*48/45 after boosting with parasite lysates. The overall IFA quality was unacceptably poor.

***Response #14****: We thank the reviewer for this insightful suggestion. Panels A of Figures 1, 2, and 3 have been removed, and the percentage of respondents has been added to the associated table. This further illustrates that the rPvs48/45 protein's conformation does not differ or change between the liquid and solid phases in the ELISA plate. Further details are also provided in the Results section page 12 lines 250-255 (MS Revised clean version).*

*Figure 4 comment was deleted.*

*Figure 5: IgG is included in current Fig 3. Specific IgM was not detectable at these dates.*

**Reviewer #2:**

This is an interesting report suggesting cross-species protection by a Plasmodium vivax antigenic protein possibly involved in transmission-blocking activity against P. falciparum.

There is one major concern with the interpretation offered. The authors confidently assert that the African sera examined would have never been exposed to any P. vivax malaria at all. This view seems to hinge on the sera being collected prior to the now well known and commonly reported presence of endemic P. vivax in all of the nations represented in their samples. This presumes the flurry of P. vivax reports out of Africa starting in about 2010 documented a new invasion of the continent and that prior to this, no P. vivax transmission occurred. This is very highly unlikely. Instead, P. vivax transmission has very probably always occurred in Africa but went unnoticed due to inherently very low parasitemias ,or due to microscopic misidentification as P. ovale. This reviewer does not accept the presumption of cross-reactive P. vivax antigen in people who very probably had experience with P. vivax infection.

The authors should consider the likelihood of the observed seropositivity being due to actual exposure to endemic *P. vivax*.

***Response #1.*** *We thank the reviewer for this valuable observation. Although it is uncertain how long P. vivax has been circulating in Africa, it is likely that even if it has been broadly circulating for a long time, it has been very low-density and would not affect the findings of this study. This has been discussed in the current version,*

*in the conclusion subsection of the Abstract, Introduction and Discussion.*
