# Supplementary material for: Cross-reactivity of rPvs48/45, a recombinant Plasmodium vivax protein, with plasma from Plasmodium falciparum endemic areas of Africa
Source: PLoS One. 2025 Mar 18;20(3):e0302605. doi: 10.1371/journal.pone.0302605 (PMC11918314; doi:10.1371/journal.pone.0302605)
Supplement: Attachment — Submitted filename: Response to reviewer letter.docx [file pone.0302605.s007.docx]

*January 24, 2025*

**Letter**

We would like to thank the editor and reviewers for their valuable comments and for giving us another opportunity to improve the Document. Please find below, point by point, our responses/suggestions to the concerns raised.

We also supply as figures those verified and suggested after downloading from the Preflight Analysis and Conversion Engine (PACE) digital diagnostic tool PACE.

**Reply to Reviewer (s)**

**Journal Requirements:

Please review your reference list to ensure that it is complete and correct. If you have cited papers that have been retracted, please include the rationale for doing so in the manuscript text, or remove these references and replace them with relevant current references. Any changes to the reference list should be mentioned in the rebuttal letter that accompanies your revised manuscript. If you need to cite a retracted article, indicate the article’s retracted status in the References list and also include a citation and full reference for the retraction notice.

**Answer: We have checked the reference list and everything seems to be correct, with no indication of retracted reference.**

**Reviewer #1**: All my previous concerns have been addressed except one pertaining to IFA data. I would suggest that authors insert a disclaimer (on lines 340 and 426-427) that the IFA reactivity to Pf may also be due to primary IgM response against immunizing Pf gametoc4yte proteins (not tested).

**Response: We thank the reviewer for the suggestions. We have now revised the relevant paragraphs to address these concerns. Cf. Manuscript with Track changes: Lines 340-344 (results section) and Lines 431-433 (discussion section)**

While revising your submission, please upload your figure files to the Preflight Analysis and Conversion Engine (PACE) digital diagnostic tool, [https://pacev2.apexcovantage.com/](https://track.editorialmanager.com/CL0/https:%2F%2Fpacev2.apexcovantage.com%2F/1/010f01946fded571-dc2b2f9d-df59-492e-a3a6-f3222d60e135-000000/jjgJxNPaztaRqIW0d0YwAdqm_slaNgp_7BdwRG8OmRA=193). PACE helps ensure that figures meet PLOS requirements. To use PACE, you must first register as a user. Registration is free. Then, login and navigate to the UPLOAD tab, where you will find detailed instructions on how to use the tool. If you encounter any issues or have any questions when using PACE, please email PLOS at [figures@plos.org](mailto:figures@plos.org). Please note that Supporting Information files do not need this step.

**Responses: All figures have now been checked with PACE to meet PLOS recommendations. The figures now supplied are those suggested through PACE.**

*Sincerely*

*Saidou Balam*
